# PANDA: Patch-based unsupervised deep learning for brain anomaly detection via age prediction in fetal MRI

**DOI:** 10.1162/IMAG.a.1293

**Published:** 2026-07-13

**Authors:** Yingqi Hao, Mingxuan Liu, Juncheng Zhu, Hongjia Yang, Haoxiang Li, Min Kang, Yan Song, Hua Lai, Xiaoling Zhou, Gang Ning, Yi Liao, Haibo Qu, Qiyuan Tian

**Affiliations:** Department of Radiology, West China Second University Hospital, Sichuan University, Chengdu, Sichuan Province, China; School of Biomedical Engineering, Tsinghua Medicine, Tsinghua University, Beijing, China; Key Laboratory of Birth Defects and Related Diseases of Women and Children, Ministry of Education, Chengdu, Sichuan Province, China; Department of Radiology, Chengdu Seventh People’s Hospital (Affiliated Cancer Hospital of Chengdu Medical College), Chengdu, China; Department of Radiology, Sichuan Provincial Women’s and Children’s Hospital, The Affiliated Women’s and Children’s Hospital of Chengdu Medical College, Chengdu, China; Chengdu Women’s and Children’s Central Hospital, School of Medicine, University of Electronic Science and Technology of China, Chengdu, China

**Keywords:** age difference, anomaly localization, ventriculomegaly, germinal matrix-intraventricular hemorrhage, subependymal cyst

## Abstract

Fetal brains frequently exhibit anomalies arising from a broad spectrum of etiologies, such as genetic, infectious, hemorrhagic, or hypoxic-ischemic insults, many of which are associated with serious clinical morbidities. Unsupervised anomaly detection, which learns exclusively from normal cases to identify significant deviations from normative patterns without prior knowledge of specific anomaly types, offers a promising approach for automatic diagnosis of such conditions. In particular, recent studies have demonstrated that the absolute age difference (AAD) between predicted gestational age (PGA) of deep learning models from MRI images and biological gestational age (BGA) shows potential for detecting fetal brain anomalies, albeit with limited performance. To enhance anomaly detection capabilities, this study introduces a three-dimensional (3D) Patch-based brain ANomaly Detection framework via Age prediction (PANDA), utilizing the maximum AAD across all patches (MaxAAD) as a biomarker for identifying fetal brain anomalies. Experiments were conducted on MRI data from a large clinical cohort of 1,316 fetuses comprising 711 normal cases and 605 abnormal cases, including 343 with ventriculomegaly (VM), 50 with germinal matrix-intraventricular hemorrhage (GMH-IVH), and 212 with subependymal cysts (SEC). PANDA achieved the best diagnostic performance with an area under the receiver operating characteristic curve (AUROC) of 0.762 and an area under the precision-recall curve (AUPR) of 0.790. Subgroup analysis across the three disease categories further revealed consistently superior performance.

## 1 Introduction

Accurate detection of fetal brain anomalies in prenatal diagnosis is essential, as these anomalies are frequent (approximately 3 in 1000 pregnancies) and associated with serious clinical adverse outcomes (Dunbar et al., 2021; Griffiths et al., 2017; Tonni et al., 2016). Compared to ultrasound imaging (USI), magnetic resonance imaging (MRI) provides superior soft tissue contrast and image spatial resolution, enabling more accurate detection of fetal brain anomalies (Faghihpirayesh et al., 2024; M. Liu, Li, et al., 2024; Sileo et al., 2022). However, current clinical practice relies on visual inspection of fetal brain MRI to detect and locate anomalies, which is subjective and prone to errors due to rapidly evolving fetal anatomy and absent normative references (Shi et al., 2020; Yun et al., 2025). Therefore, developing an automated quantitative framework to identify anomalies in fetal brain is essential for improving diagnostic accuracy and efficiency.

Recent advancements in deep learning have introduced automated solutions for detecting fetal brain anomalies, showing promise in reducing inter-observer variability and improving diagnostic accuracy and efficiency. Most existing methods rely on supervised learning paradigms, which utilize annotated datasets to train models for specific classification or segmentation tasks (Ahmad, 2026; Kumar & Kumar Goel, 2022; Lo et al., 2022; Mushtaq & Veningston, 2025). For example, Chowdhury et al. proposed StackFBAs, which utilizes a stacking strategy to integrate multiple convolutional neural networks (CNNs) with a k-nearest neighbors (KNN) meta-classifier for accurate classification of fetal brain abnormalities (Chowdhury et al., 2023). Similarly, Vahedifard et al. developed a U-Net-based deep learning model to segment brain tissues and automatically measure lateral ventricles for the detection of ventriculomegaly (Vahedifard et al., 2023). However, the success of these supervised methods is heavily dependent on the availability of large-scale, high-quality, and annotated datasets (M. Liu, Jiao, et al., 2024; Meshaka et al., 2022). For fetal brain MRI, such datasets are scarce due to the rarity of certain pathologies and the high cost of expert annotation. For instance, even germinal matrix-intraventricular hemorrhage (GMH-IVH), recognized as the most common type of fetal brain hemorrhage, has an estimated incidence of only 0.5–0.9 per 1000 pregnancies (Sanapo et al., 2017). As a result, this data scarcity makes it challenging to train robust deep learning models for detecting a wide range of rare fetal brain anomalies and limits the generalization capability of supervised models.

To overcome the reliance on large-scale labeled datasets, deep learning-based fetal brain age prediction has been used for unsupervised detection of fetal abnormalities (Everwijn et al., 2021, 2019; Gong et al., 2021; Roibu et al., 2023; Shi et al., 2020; Yun et al., 2025), since gestational age is routinely established prior to fetal MRI as part of standard clinical care and does not require additional annotation. However, existing methods still demonstrate suboptimal performance. For instance, one study predicted gestational age using the center slice of the brain and employed the absolute age difference (AAD) between predicted gestational age (PGA) and biological gestational age (BGA) as a biomarker for diagnosing small head circumference (SHC), malformations (MF), and ventriculomegaly (VM) (Shi et al., 2020). However, the method achieved only 0.66 area under the receiver operating characteristic curve (AUROC) for VM diagnosis on a small number of abnormal fetuses (*n* = 46). Yun et al. further improved this approach by using multiple slices as input to detect a single abnormality type, achieving an AUROC of 0.69 for VM diagnosis (Hong et al., 2021; Yun et al., 2025), yet its performance on other abnormalities remains unverified. Additionally, these methods are based on 2-D slices or stacks, thereby limiting the effective utilization of inter-slice contextual information.

To improve unsupervised fetal brain anomaly detection, this study proposes a three-dimensional (3D) Patch-based unsupervised deep learning framework for brain ANomaly Detection via Age prediction (PANDA), which utilizes the maximum AAD across all patches (MaxAAD) as a biomarker for identifying fetal brain abnormalities. Since patches in pathological regions exhibit larger AAD than normal regions, MaxAAD not only improves anomaly detection performance but also enables anomaly localization. Extensive experiments on a large cohort (*n* = 1,316) of fetal brain MRI data, which includes three types of anomalies—VM (Alluhaybi et al., 2022), subependymal cysts (SEC) (Yang et al., 2017), and GMH-IVH (Parodi et al., 2020), demonstrate that the proposed PANDA framework achieves the best performance.

## 2 Materials and Methods

### 2.1 Data collection

Experiments were performed with the approval of the Institutional Review Board of West China Second University Hospital, Sichuan University, Chengdu Women’s and Children’s Central Hospital, and Sichuan Provincial Women’s and Children’s Hospital, and in accordance with the principles outlined in the Declaration of Helsinki. MRI data from 1,756 pregnant women (22–36 weeks gestation) undergoing prenatal examinations were retrospectively used for training and internal validation. All image stacks were acquired consecutively along axial, coronal, and sagittal orientations on one 3.0-T (Siemens MAGNETOM Skyra) and two 1.5-T (Philips Achieva and United Imaging uMR 570) scanners from October 2015 to October 2023. Cases included 1,151 normal and 605 abnormal fetuses (343 with VM, 50 with GMH-IVH, and 212 with SEC). The BGA was determined as the time from the first day of the last menstruation and confirmed with a crown-rump length measurement at the first trimester USI. Data from 440 normal cases were used for brain age prediction training and hyperparameter tuning. Data from the remaining 711 normal and 605 abnormal cases were used for performance evaluation. For external validation, data were retrospectively collected from Chengdu Women’s and Children’s Central Hospital and Sichuan Provincial Woman’s and Children’s Hospital using one 1.5-T (GE Signa HDxt) scanner and one 3.0-T (Siemens MAGNETOM Vida) scanner, comprising 48 typically developing fetuses and 14 fetuses diagnosed with GMH-IVH.

### 2.2 Fetal MRI acquisition protocol

To minimize image artifacts caused by fetal motion, 2D imaging sequences were used to acquire T_2_-weighted MR images. For the 3.0-T Siemens MAGNETOM Skyra and Vida MRI scanners, a half-Fourier-acquired single-shot turbo spin-echo (HASTE) sequence was used. For the 1.5-T United Imaging uMR 570 MRI and GE Signa HDXt scanners, a single shot fast spin echo (SSFSE) was used. For the 1.5-T Philips Achieva MRI scanner, a turbo spin echo single shot (TSE) sequence was used. Imaging section thickness was 3.0–9.1 mm, without spacing between sections. Image in-plane spatial resolution was 1 × 1 mm².

### 2.3 Inclusion and exclusion criteria of fetuses

Inclusion criteria for normal fetuses were: (i) singleton pregnancies with no maternal medical conditions (including nicotine or substance dependence, morbid obesity, cancer, diabetes, and gestational diabetes); and (ii) maternal age between 18 and 45 years. Exclusion criteria were: (i) any pregnancy or fetal anomalies, including congenital infections, multiple gestations, fetal anomalies in the brain or other organs, and chromosomal aberrations; (ii) any contraindications to MRI; and (iii) 3D volumes with reconstruction failure, severe artifacts, tissue incompleteness, or extremely low contrast after volumetric reconstruction using the NeSVoR method, as assessed by an expert radiologist.

Inclusion criteria for fetuses with anomalies including VM, GMH-IVH, or SEC were: (i) singleton pregnancies with confirmed fetal pathology via MRI; and (ii) maternal age between 18 and 45 years, without known maternal medical conditions (including nicotine or substance dependence, morbid obesity, cancer, diabetes, and gestational diabetes). Exclusion criteria were: (i) any additional pregnancy or fetal anomalies, including congenital infections, anatomical anomalies in the brain or other organs, and chromosomal aberrations; (ii) any contraindications to MRI; and (iii) 3D volumes with reconstruction failure, severe artifacts, tissue incompleteness, or extremely low contrast after NeSVoR volumetric reconstruction, as evaluated by an expert radiologist.

The severity of internal GMH-IVH data was evaluated and graded using a 1-4 grading scale (Epstein et al., 2021; Papile et al., 1978) by an expert radiologist, with Grade I for subependymal hemorrhage (*n* = 5), Grade II for intraventricular hemorrhage filling <50% of the ventricle with ventricular size of ≤15 mm (*n* = 29), Grade III for intraventricular hemorrhage filling >50% of the ventricle with a ventricular size of ≥15 mm (*n* = 8), and Grade IV for the presence of parenchymal hemorrhage (*n* = 8).

### 2.4 PANDA framework

**Fig. 1.**
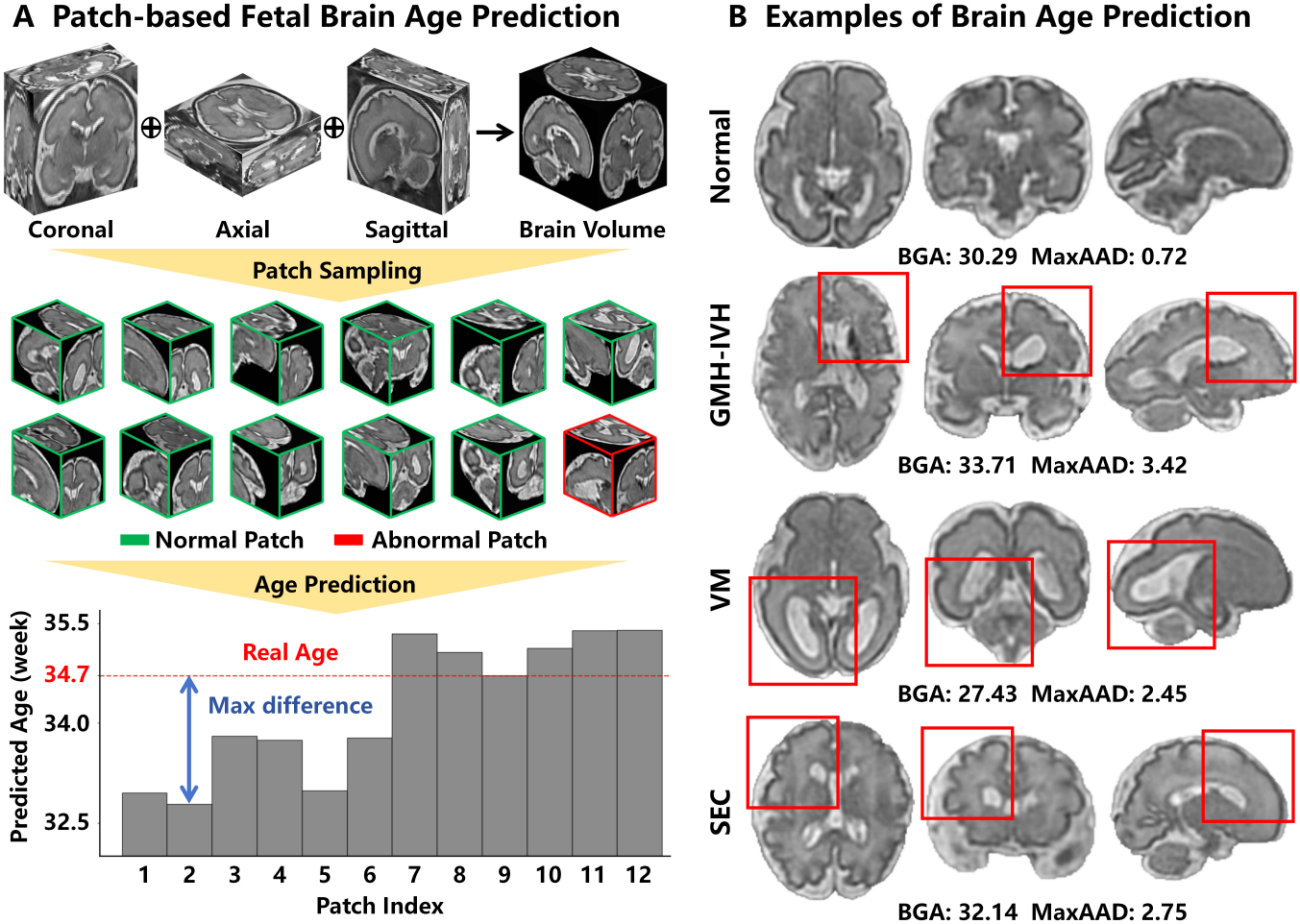
Patch-based fetal brain age prediction. (A) For each fetus, a 3D brain volume is reconstructed from multi-planar thick-slice stacks and divided into 8-12 overlapping cubic patches. Each patch, consisting of 64 × 64 × 64 voxels, is then input into a 3D CNN pre-trained on data of normal fetuses for predicting gestational age. The maximum absolute age difference (MaxAAD) between the predicted and biological gestational age across all patches serves as a biomarker for brain anomaly detection. (B) Exemplary brain images from a normal fetus and three representative fetuses with anomalies, that is, ventriculomegaly (VM), germinal matrix-intraventricular hemorrhage (GMH-IVH), and subependymal cysts (SEC), are displayed, with red boxes highlighting patches with the largest MaxAAD and abnormal areas.

PANDA trains a deep learning model using normal fetuses to obtain PGA from each brain volume patch and uses the largest difference compared to BGA across all patches, i.e., MaxAAD, as a biomarker for fetal brain anomalies (Fig. 1A). Specifically, 3D brain volumes at 0.8 × 0.8 × 0.8 mm³ isotropic resolution were first reconstructed from multi-planar thick-slice stacks using NeSVoR, an implicit neural representation-based slice-to-volume reconstruction (SVR) method (Xu et al., 2023). Second, each brain volume was divided into 8-12 overlapping cubic patches of size 64 × 64 × 64 voxels. During training, a 3D Simple Fully Convolutional Network (SFCN) (Peng et al., 2021) was trained for predicting age from each patch using data of normal fetuses. During inference, a gestational age, $PGA\left(p_{i}\right)$, was predicted for each patch $p_{i}$ to calculate its AAD compared to the BGA, BGA . The patch with the maximum deviation is identified as:

$$i^{*}=\arg\underset{i}{\max}\left|PGA\left(p_{i}\right)-BGA\right|,$$

and the corresponding MaxAAD is defined as:

$$\text{MaxAAD}=\left|PGA\left(p_{i^{*}}\right)-BGA\right|,$$

where $PGA\left(p_{i^{*}}\right)\;$ is the worst PGA with the largest deviation from BGA.

In the PANDA framework, 3D patches were extracted using the following procedure. First, the minimal bounding box encompassing the brain mask was identified:

$$B_{brain}=\left[x_{min},x_{max},y_{min},y_{max},z_{min},z_{max}\right].$$

Next, the number of patches along each dimension was computed as:

$$n_{x}=\left\lceil \frac{L_{x}}{s}\right\rceil ,\;n_{y}=\left\lceil \frac{L_{y}}{s}\right\rceil ,\;n_{z}=\left\lceil \frac{L_{z}}{s}\right\rceil ,$$

where $L_{x}\;=x_{max}-x_{min}+1$ , $L_{y}\;=y_{max}-y_{min}+1$ , $L_{z}=z_{max}-z_{min}+1L_{z}=z_{max}-z_{min}+1$ represent brain dimensions, s = 64 denotes patch size. The starting and ending positions for patch distribution were defined as:

$$x_{start}=x_{min},\;x_{end}=x_{max}-s+1;$$

$$y_{start}=y_{min},\;y_{end}=y_{max}-s+1;$$

$$z_{start}=z_{min},\;z_{end}=z_{max}-s+1.$$

Patch starting positions were uniformly distributed using:

$$x_{indices}=\text{linspace}\left(x_{start},x_{end},n_{x}\right),$$

$$y_{indices}=\text{linspace}\left(y_{start},y_{end},n_{y}\right),$$

$$z_{indices}=\text{linspace}\left(z_{start},z_{end},n_{z}\right).$$

Finally, each patch $P_{i,j,k}$ was defined by its 3D coordinates:

$$P_{i,j,k}=\left[x_{i},x_{i}+s-1,y_{j},y_{j}+s-1,z_{k},z_{k}+s-1\right],$$

where $i\in \left[0,n_{x}-1\right],j\in \left[0,n_{y}-1\right]$, $k\in \left[0,n_{z}-1\right]$, resulting in a total of $n_{x}\times n_{y}\times n_{z}$ overlapping patches (typically 8 or 12 patches per fetal brain volume).

### 2.5 Comparison methods

The proposed PANDA method was compared to three state-of-the-art (SOTA) methods that predict PGA in different ways for deriving AAD:

1. 2D Single-slice AAD: a 2D ResNet101 (He et al., 2016) was trained using a single central slice of the brain volume (Shi et al., 2020);
2. 2D Multi-slice AAD: a 2D ResNet101 (He et al., 2016) was trained using multiple central slices along each acquisition orientation (i.e., axial, coronal, and sagittal), with the PGA determined as the mode of multiple predictions from four central slices of each stack (Yun et al., 2025);
3. 3D Whole-brain AAD: a 3D SFCN was trained using the whole-brain volume (Peng et al., 2021);
4. 3D Patch MeanAAD: the mean value of PGAs across all patches, $\bar{PGA}$, was used to derive the MeanAAD as:
  $$\text{MeanAAD}=\left|\bar{PGA}-BGA\right|$$.
  To further validate the advantage of PANDA under limited label availability, we compared it against supervised binary classification models. Specifically, two supervised baselines were trained on 440 normal cases and 50 labeled GMH-IVH cases from the internal dataset. All models were then evaluated on the external validation dataset:
5. Supervised SFCN (w/o age): a 3D SFCN-based binary classifier trained on whole-brain volumes without incorporating age information;
6. Supervised SFCN (w/ age): a 3D SFCN-based binary classifier with gestational age encoded via positional encoding and concatenated with image features as an additional input.

### 2.6 Training details

To ensure a fair comparison, PANDA and comparison unsupervised methods utilized the same training dataset and configuration, including the optimizer, batch size, learning rate, and number of epochs. Specifically, MRI data from 440 normal fetuses were used for model development, with 400 allocated for training and 40 for selecting the best checkpoint. All models were trained on an NVIDIA A800 GPU. The AdamW optimizer was configured with a learning rate of 0.0001, betas of 0.9 and 0.999, a weight decay of 10^-5^, and a cosine annealing learning rate scheduler. The network was trained for 1000 epochs with a batch size of 32, using L_2_ Loss as the loss function.

For the supervised baselines, training was also performed on an NVIDIA A800 GPU. The AdamW optimizer was configured with a learning rate of 0.0001, betas of 0.9 and 0.999, and a weight decay of 10^-5^. The network was trained for 1000 epochs with a batch size of 4, using the cross-entropy loss function.

### 2.7 Statistical analysis

Five quantitative metrics were adopted for evaluating and comparing the anomaly detection performance of different methods: (1) AUROC; (2) area under the precision-recall curve (AUPR); (3) sensitivity at 0.70 specificity; and (4) specificity at 0.70 sensitivity; (5) F1 Score. Confidence intervals (CIs) for AUROC, AUPR, and F1 Score were calculated via bootstrap (1000 resamples). CIs for sensitivity and specificity were determined using Wilson score intervals. Statistical comparisons employed the DeLong test for AUROC, bootstrap method for AUPR and F1 Score, and McNemar test for model performance at specific operating points.

### 2.8 Ablation study

Three ablation studies were conducted to investigate the impact of key design choices in the PANDA framework, including the model architecture, input patch size, and the aggregation strategy used to derive the biomarker:

1. The effect of model architecture was evaluated by comparing three representative networks: (i) SFCN, which serves as the default backbone in PANDA; (ii) a Vision Transformer (ViT) (Dosovitskiy et al., 2020); and (iii) a Visual State Space Model (V-Mamba) (Y. Liu et al., 2024). All three architectures were trained and evaluated under identical settings, including a patch size of 64 and the MaxAAD aggregation strategy.
2. The influence of input patch size was examined by varying the patch dimensions among 32 × 32 × 32, 64 × 64 × 64, and 96 × 96 × 96 voxels while keeping the model architecture (SFCN) and the MaxAAD aggregation strategy fixed. This experiment aims to investigate the trade-off between localization precision and contextual information across different patch sizes.
3. The aggregation strategy for computing the biomarker was investigated to assess whether averaging over multiple high-AAD patches could improve the results. Instead of using only the single patch with the maximum AAD (i.e., MaxAAD), we evaluated top-*k* aggregation strategies, in which the *k* largest AAD values across all patches were averaged to derive the biomarker. Specifically, for a patch size of 64 × 64 × 64 (yielding 8–12 patches per volume), three aggregation levels were compared (i.e., max, top-2, and top-4). For a patch size of 32 × 32 × 32 (yielding 27–96 patches per volume), six aggregation levels were compared (i.e., max, top-2, top-4, top-8, top-16, and top-32).

## 3 Results

### 3.1 Dataset characteristics

For internal validation, no significant group differences in BGA were found between normal and abnormal fetuses. Significant group differences in maternal age (MA) and magnetic field strength were found between normal and abnormal fetuses (*P* < 0.05). Additionally, among the three subgroups of abnormal fetuses, significant differences were found in BGA and magnetic field strength (*P* < 0.05), but not found in MA. For external validation, no significant group differences were found for any variables (Table 1).

**Table 1.** Demographic information and statistical comparisons by group.

	Internal validation							External validation		
				Abnormal (*n* = 605)						
Demographic	Normal (*n* = 711)	Abnormal (*n* = 605)	*P* Value	VM (*n* = 343)	SEC (*n* = 212)	GMH-IVH (*n* = 50)	*P* Value	Normal (*n* = 48)	Abnormal (*n* = 14)	*P* Value
BGA (wk)	30.60 ± 3.29	30.87 ± 3.06	0.117	30.42 ± 2.96	32.20 ± 2.62	28.36 ± 3.10	< 0.001	31.40 ± 2.82	30.04 ± 3.03	0.123
MA (y)	29.42 ± 3.81	28.93 ± 4.06	0.041	28.91 ± 4.16	29.20 ± 3.82	27.85 ± 4.26	0.121	NA	NA	NA
Magnetic field strength			< 0.001				< 0.001			0.759
1.5 T	670	604		343	212	49		19	7	
3.0 T	41	1		0	0	1		29	7	

Continuous data are expressed as means ± SDs and categorical data as numbers. GA and MA were compared using *t* test or analysis of variance. Magnetic fields for fetal MRI examination (1.5 T vs. 3.0 T) were compared using Fisher exact test.

BGA = biological gestational age; MA = maternal age; VM = ventriculomegaly; SEC = subependymal cysts; GMH-IVH = germinal matrix-intraventricular hemorrhage.

### 3.2 Qualitative results of anomaly detection

**Fig. 2.**
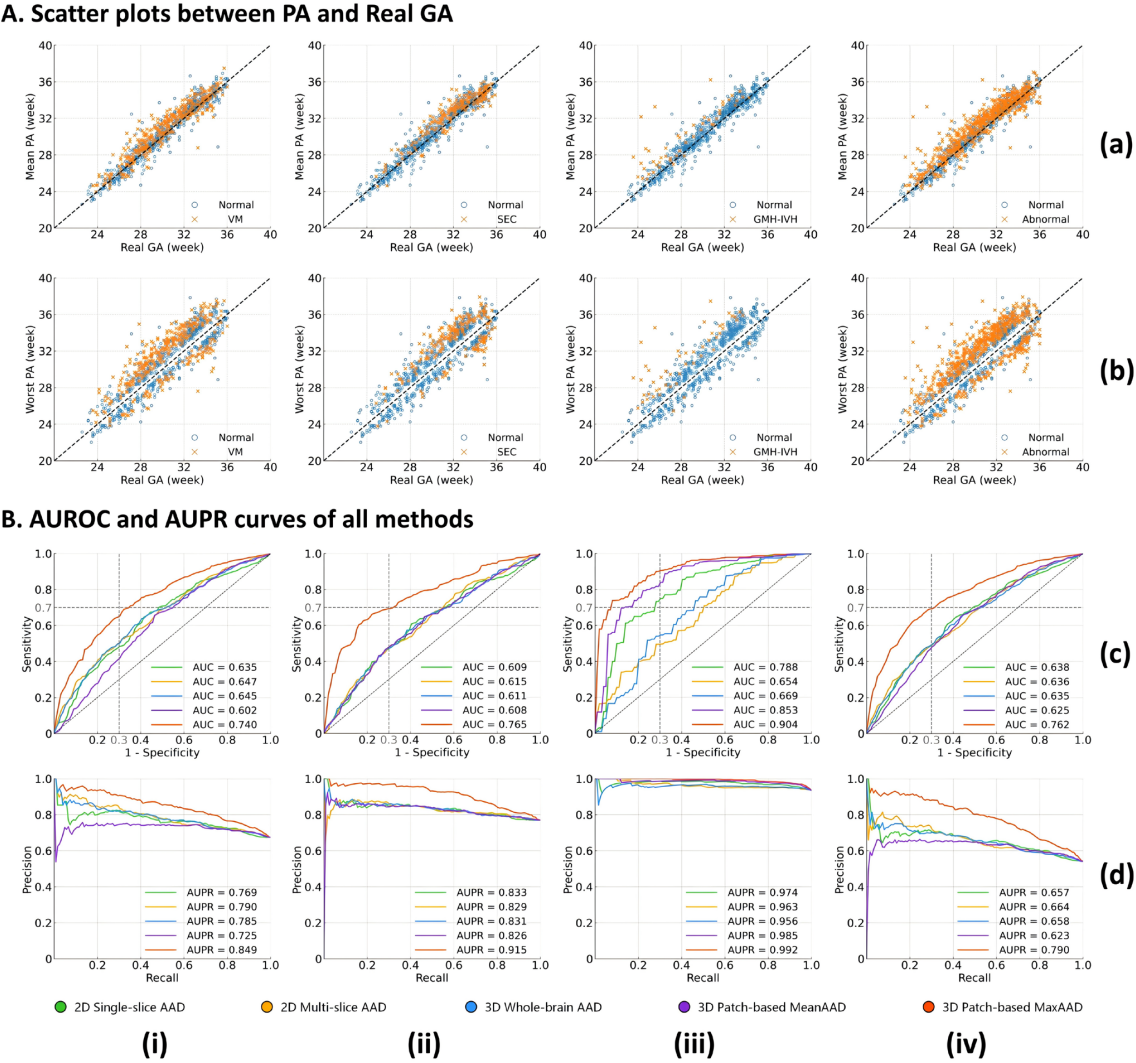
Abnormality detection performance quantification. (A) Scatter plots of biological gestational age (BGA) versus (a) the mean value of predicted GAs across all patches (i.e., average PGA) and (b) the PGA with the largest difference compared to the BGA (i.e., worst PGA) are displayed with 45 degree diagonal line, for differentiating normal fetuses (blue circles) and fetuses with abnormalities (yellow crosses), including (i) ventriculomegaly (VM), (ii) subependymal cysts (SEC), (iii) germinal matrix-intraventricular hemorrhage (GMH-IVH), and (iv) all three anomalies. (B) The curves for the (c) areas under the receiver operating characteristic (AUROC) and (d) areas under the precision-recall (AUPR) are displayed for all fetal brain anomaly detection methods.

**Fig. 3.**
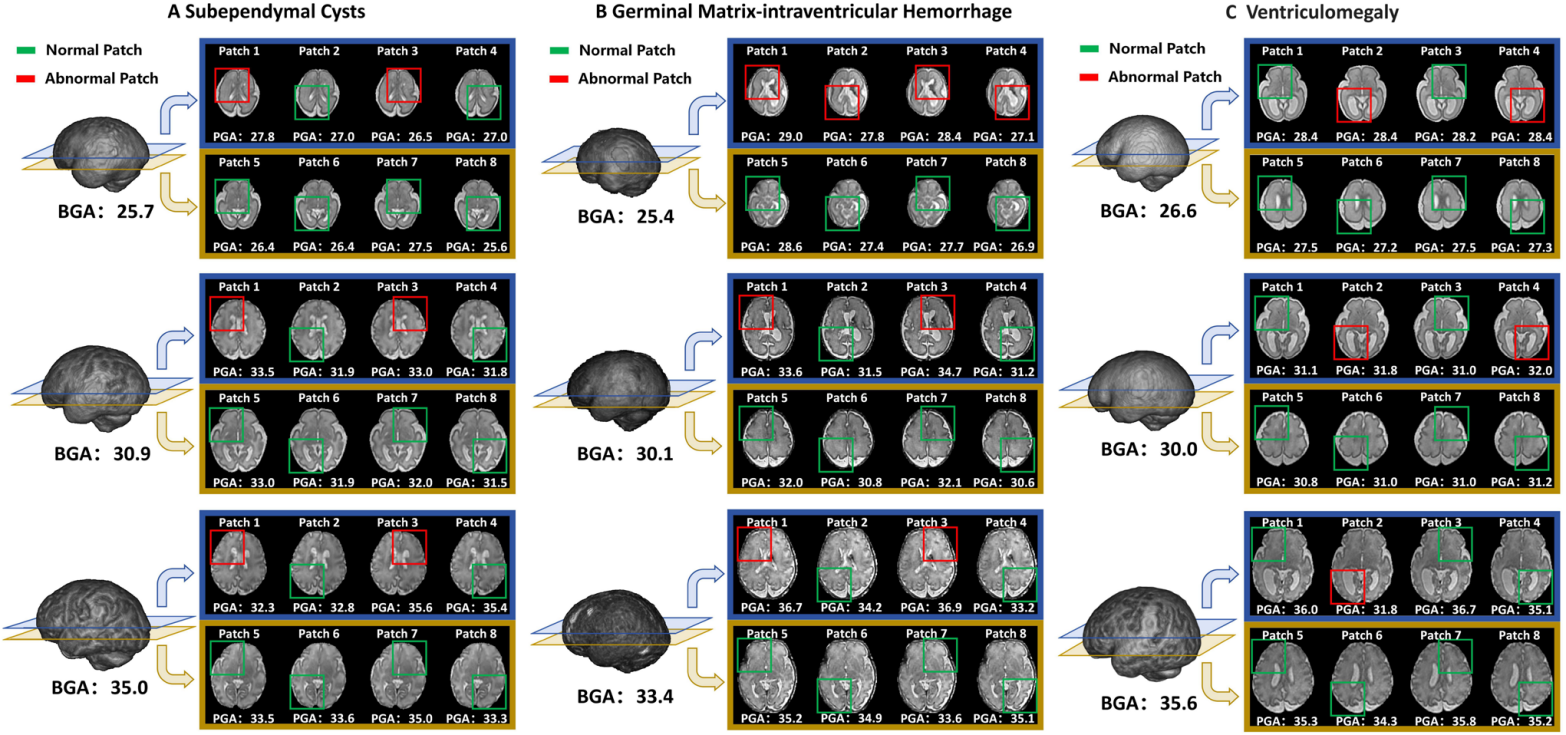
Patch-wise predicted gestational ages. Both normal patches (green boxes) and abnormal patches (red boxes), including (A) subependymal cysts (SEC), (B) germinal matrix–intraventricular hemorrhage (GMH-IVH), and (C) ventriculomegaly (VM) are shown with their biological and patch-wise predicted gestational ages.

Qualitatively, PANDA produced higher AAD for abnormal patches and lower AAD for normal patches (Fig. 1B). Compared with MeanAAD, MaxAAD yielded markedly improved visual separability in the scatter plot (Fig. 2A), thereby supporting superior effectiveness for diagnosing anomalies. Furthermore, visualization results demonstrate that in abnormal brains, patches encompassing pathological regions, including SECs, hemorrhagic lesions (GMH-IVH), and dilated ventricles (VM), exhibited higher AAD (Fig. 3), indicating the potential of PANDA for lesion localization compared to traditional age prediction-based anomaly detection methods.

### 3.3 Quantitative results of anomaly detection

Quantitative analyses further substantiate that PANDA achieved the best performance in detecting fetal brain anomalies (Fig. 2B, Table 2, Table 3). Specifically, PANDA achieved an AUROC of 0.762, AUPR of 0.790, sensitivity of 0.692, specificity of 0.686 and F1 score of 0.741 for anomaly detection, significantly outperforming other methods (*P* < 0.05). Subgroup analysis demonstrates that PANDA attained an AUROC of 0.740, AUPR of 0.849, sensitivity of 0.651, specificity of 0.656, and F1 score of 0.819 for VM diagnosis; an AUROC of 0.765, AUPR of 0.915, sensitivity of 0.692, specificity of 0.679, and F1 score of 0.874 for SEC diagnosis; and an AUROC of 0.904, AUPR of 0.992, sensitivity of 0.906, specificity of 0.920, and F1 score of 0.971 for GMH-IVH diagnosis. Except for the AUPR and specificity metrics in GMH-IVH diagnosis, PANDA significantly outperformed other methods across all evaluated metrics (*P* < 0.05, Table 4).

**Table 2.**
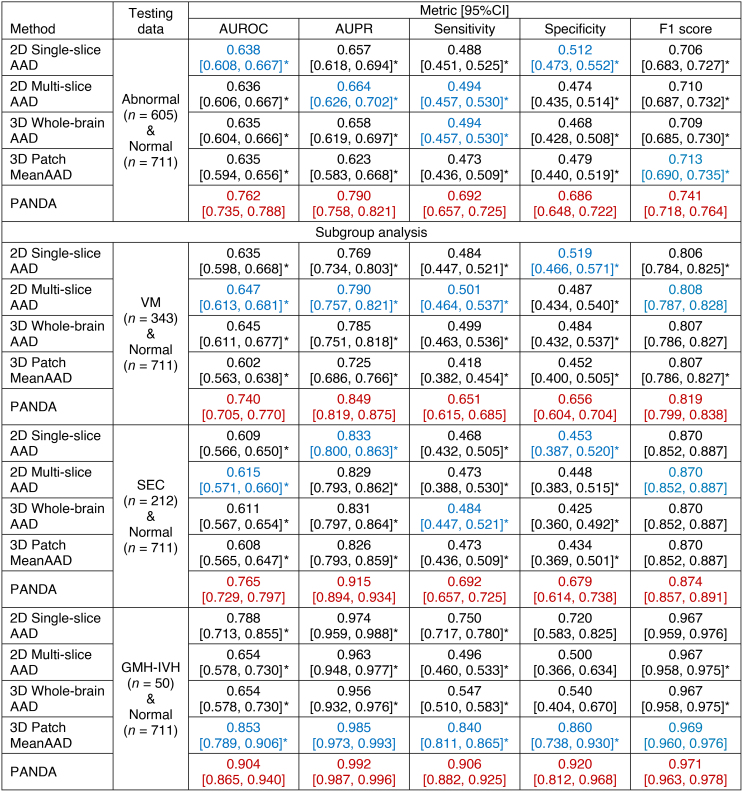
Anomaly detection performance (internal validation).

The AUROC, AUPR, F1 Score and performance at fixed sensitivity and specificity thresholds (0.70) are listed for PANDA and all methods for comparison. An asterisk (*) in the table indicates a statistically significant difference (*P* < 0.05) compared to PANDA. The values in square brackets in the tables represent confidence intervals (CIs). The red and blue text highlight the highest and second highest scores.

VM = ventriculomegaly; SEC = Subependymal Cysts; GMH-IVH = germinal matrix-intraventricular hemorrhage; AUROC = area under the receiver operating characteristic curve; AUPR = area under the precision recall curve.

**Table 3.**
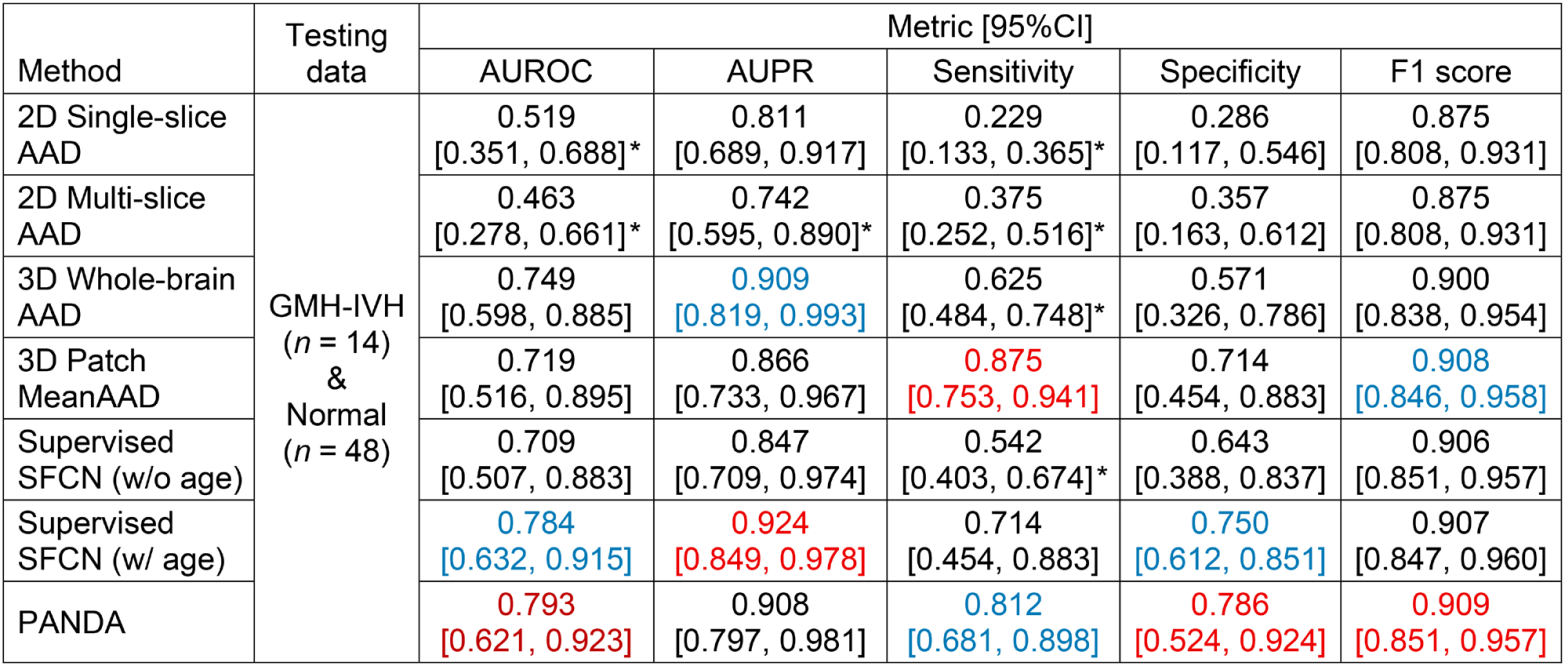
Anomaly detection performance (external validation).

The AUROC, AUPR, F1 Score and performance at fixed sensitivity and specificity thresholds (0.70) are listed for PANDA and all methods for comparison. An asterisk (*) in the table indicates a statistically significant difference (*P* < 0.05) compared to PANDA. The values in square brackets in the tables represent 95% confidence intervals (CIs). The red and blue text highlight the highest and second highest scores.

VM = ventriculomegaly; SEC = Subependymal Cysts; GMH-IVH = germinal matrix-intraventricular hemorrhage; AUROC = area under the receiver operating characteristic curve; AUPR = area under the precision recall curve.

**Table 4.** Significance levels of diagnostic accuracy.

Method	AUROC	AUPR	Sensitivity	Specificity	F1 score
Internal anomaly detection					
2D Single-slice AAD	< 0.001	< 0.001	< 0.001	< 0.001	< 0.001
2D Multi-slice AAD	< 0.001	< 0.001	< 0.001	< 0.001	< 0.001
3D Whole-brain AAD	< 0.001	< 0.001	< 0.001	< 0.001	< 0.001
3D Patch MeanAAD	< 0.001	< 0.001	< 0.001	< 0.001	< 0.001
Internal VM detection					
2D Single-slice AAD	< 0.001	< 0.001	< 0.001	0.012	0.004
2D Multi-slice AAD	< 0.001	0.011	< 0.001	0.001	0.052
3D Whole-brain AAD	< 0.001	0.004	< 0.001	0.001	0.140
3D Patch MeanAAD	< 0.001	< 0.001	< 0.001	< 0.001	0.004
Internal SEC detection					
2D Single-slice AAD	< 0.001	< 0.001	< 0.001	0.008	0.308
2D Multi-slice AAD	< 0.001	< 0.001	< 0.001	< 0.001	0.316
3D Whole-brain AAD	< 0.001	< 0.001	< 0.001	< 0.001	0.310
3D Patch MeanAAD	< 0.001	< 0.001	< 0.001	< 0.001	0.310
Internal GMH-IVH detection					
2D Single-slice AAD	0.001	0.008	< 0.001	0.724	0.162
2D Multi-slice AAD	< 0.001	< 0.001	< 0.001	1.000	0.038
3D Whole-brain AAD	< 0.001	< 0.001	< 0.001	0.208	0.044
3D Patch MeanAAD	0.007	0.137	< 0.001	0.010	0.408
External GMH-IVH detection					
2D Single-slice AAD	0.001	0.058	< 0.001	0.052	0.074
2D Multi-slice AAD	0.003	0.016	< 0.001	0.442	0.102
3D Whole-brain AAD	0.610	0.995	0.031	1.000	0.880
3D Patch MeanAAD	0.211	0.076	0.453	0.031	1.000
Supervised SFCN (w/o age)	0.387	0.424	0.007	0.189	1.000
Supervised SFCN (w/ age)	0.931	0.644	0.607	1.000	1.000

The values represent the significance levels (*P*-values) for differences in performance metrics between PANDA (3D Patch MaxAAD) and other methods in internal and external validation. Comparisons are shown for the overall dataset and for subgroup analysis (VM, SEC, GMH-IVH).

VM = ventriculomegaly; SEC = Subependymal Cysts; GMH-IVH = germinal matrix-intraventricular hemorrhage; AUROC = area under the receiver operating characteristic curve; AUPR = area under the precision recall curve.

For external GMH-IVH detection, PANDA achieved an AUROC of 0.793, an AUPR of 0.908, a sensitivity of 0.812, a specificity of 0.786, and an F1 score of 0.909, maintaining the best or near-best performance across all methods (Table 3 and Table 4). Importantly, PANDA achieved higher AUROC, sensitivity, specificity, and F1 score compared with both supervised baselines, despite not requiring anomaly labels during training.

### 3.4 Robustness to scanner and field strength variability

The distribution of MaxAAD across magnetic field strengths was examined to assess the robustness of PANDA to scanner variability (Table 5). For internal validation, normal fetuses scanned at 3.0 T exhibited a slightly higher MaxAAD (1.79 ± 1.07 weeks) than those at 1.5 T (1.36 ± 0.73 weeks), likely due to the imbalanced distribution of field strengths in the training data. For external validation, the difference between field strengths in normal fetuses was largely diminished (1.5 T: 2.16 ± 0.57 weeks vs. 3.0 T: 2.04 ± 1.02 weeks), while MaxAAD for GMH-IVH remained slightly elevated at both strengths (1.5 T: 3.70 ± 1.14 weeks vs. 3.0 T: 3.10 ± 2.06 weeks).

**Table 5.** Distribution of MaxAAD across magnetic field strengths.

		All		1.5 T		3.0 T	
Group		*n*	MaxAAD	*n*	MaxAAD	*n*	MaxAAD
Internal validation	Normal	711	1.37 ± 0.75	670	1.36 ± 0.73	41	1.79 ± 1.07
	VM	343	2.04 ± 0.93	343	2.04 ± 0.93	0	NA
	SEC	212	2.05 ± 0.82	212	2.05 ± 0.82	0	NA
	GMH-IVH	50	2.96 ± 1.75	49	2.97 ± 1.77	1	2.67 ± 0.00
External validation	Normal	48	2.09 ± 0.86	19	2.16 ± 0.57	29	2.04 ± 1.02
	GMH-IVH	14	3.40 ± 1.63	7	3.70 ± 1.14	7	3.10 ± 2.06

MaxAAD = the maximum absolute difference between the BGA and predicted GA (PGA) across all patches; VM = ventriculomegaly; SEC = Subependymal Cysts; GMH-IVH = germinal matrix-intraventricular hemorrhage.

### 3.5 Performance stratified by gestational age

Fetal brains with anomalies exhibited consistently higher MaxAAD compared to normal brains across all BGA ranges (Table 6). Specifically, fetuses were categorized into three groups based on BGA (≤ 27 weeks, between 27 and 32 weeks, and ≥ 32 weeks) to investigate the robustness of PANDA across different developmental stages (White et al., 2010). In the early gestation group (≤ 27 weeks), the MaxAAD for abnormal fetuses was 2.21 ± 1.59 weeks, higher than the 1.07 ± 0.53 weeks observed in normal fetuses. This distinction persisted (Abnormal: 2.18 ± 0.96 weeks vs. Normal: 1.37 ± 0.81 weeks) in the middle gestation group (27–32 weeks). In the late gestation group (≥ 32 weeks), the difference remained distinguishable but narrowed (Abnormal: 2.03 ± 0.82 weeks vs. Normal: 1.51 ± 0.75 weeks).

**Table 6.** Distribution of MaxAAD across biological gestational age groups for internal validation datasets.

	Normal		Abnormal	
BGA	*n*	MaxAAD	*n*	MaxAAD
≤ 27 weeks	124	1.07 ± 0.53	82	2.21 ± 1.59
27-32 weeks	297	1.37 ± 0.81	279	2.18 ± 0.96
≥ 32 weeks	290	1.51 ± 0.75	244	2.03 ± 0.82

BGA = biological gestational age; MaxAAD = the maximum absolute difference between the BGA and predicted GA (PGA) across all patches.

**Fig. 4.**
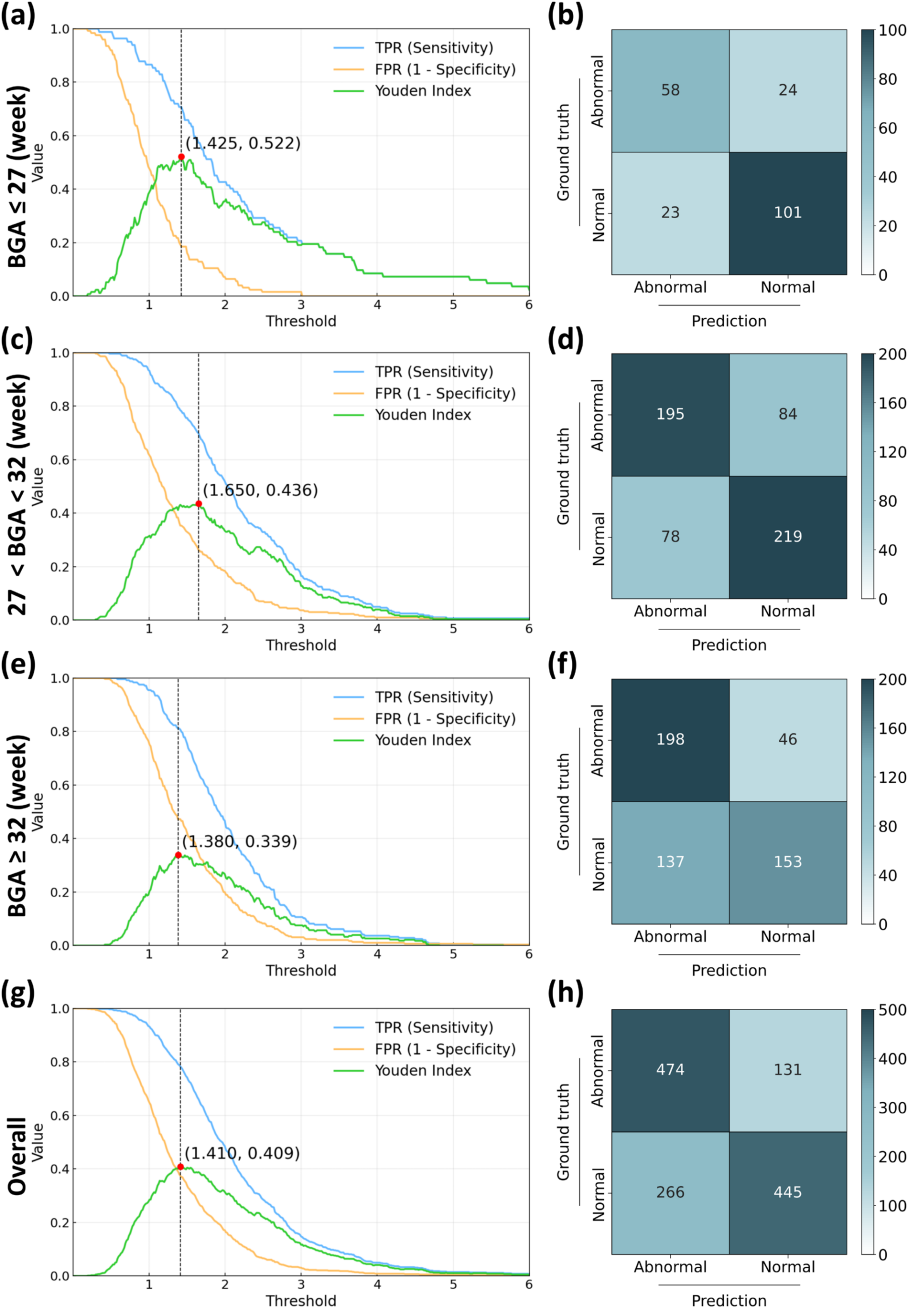
Threshold optimization and classification performance. The optimal MaxAAD thresholds were determined using the Youden Index on the testing dataset. The left column displays the threshold curves (TPR, FPR, and Youden Index), while the right column shows the corresponding confusion matrices. The results are stratified by gestational age: (a, b) the subset with BGA ≤ 27 weeks; (c, d) the subset with 27 < BGA < 32 weeks; (e, f) the subset with BGA ≥ 32 weeks; and (g, h) the overall dataset covering all anomalies. TPR = true positive rate; FPR = false positive rate.

Notably, the MaxAAD in normal fetuses showed an increasing trend with advancing BGA. This pattern may be attributed to the increased complexity and individual variability of cortical folding during the late trimester, which poses a greater challenge for precise age prediction in normal brains. Conversely, the MaxAAD in abnormal fetuses remained elevated but exhibited a slight decrease in the late gestation group. To address these developmental variations, in practice, BGA-specific thresholds for anomaly detection must be established (Fig. 4).

### 3.6 Results of ablation study

**Fig. 5.**
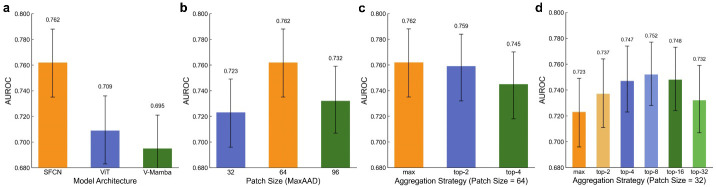
Results of Ablation Studies. The AUROC (with 95% CIs) of three ablation studies are shown. (a) Comparison of model architectures among SFCN, ViT, and V-Mamba. (b) Comparison of input patch sizes (i.e., 32, 64, and 96) using MaxAAD. (c) Comparison of aggregation strategies (i.e., max, top-2, and top-4) with a patch size of 64. (d) Comparison of aggregation strategies (i.e., max, top-2, top-4, top-8, top-16, and top-32) with a patch size of 32.

Ablation studies on model architecture, input patch size, and aggregation strategy confirm that the default PANDA configuration (SFCN backbone, patch size 64, and MaxAAD aggregation) yields the best overall performance (Fig. 5). For model architecture (Fig. 5a), SFCN achieved the highest overall AUROC of 0.762, outperforming ViT (0.709) and V-Mamba (0.695). SFCN also attained higher brain age prediction accuracy (Table 9) on normal fetuses (*R²* = 0.922 vs. *R²* = 0.902 for ViT and 0.887 for V-Mamba). For input patch size (Fig. 5b), a patch size of 64 yielded the highest AUROC of 0.762, compared to 0.732 for a patch size of 96 and 0.723 for a patch size of 32. For aggregation strategy with a patch size of 64 (Fig. 5c), MaxAAD achieved the highest AUROC of 0.762, followed by top-2 (0.759) and top-4 (0.745). With a patch size of 32 (Fig. 5d), MaxAAD achieved an AUROC of 0.723. As the aggregation number increased, AUROC first rose then declined, peaking at top-8 (0.752). Nevertheless, the best result with patch size 32 (AUROC = 0.752) remained below the default configuration (AUROC = 0.762).

### 3.7 Differentiation of anomaly types and severity grading

**Fig. 6.**
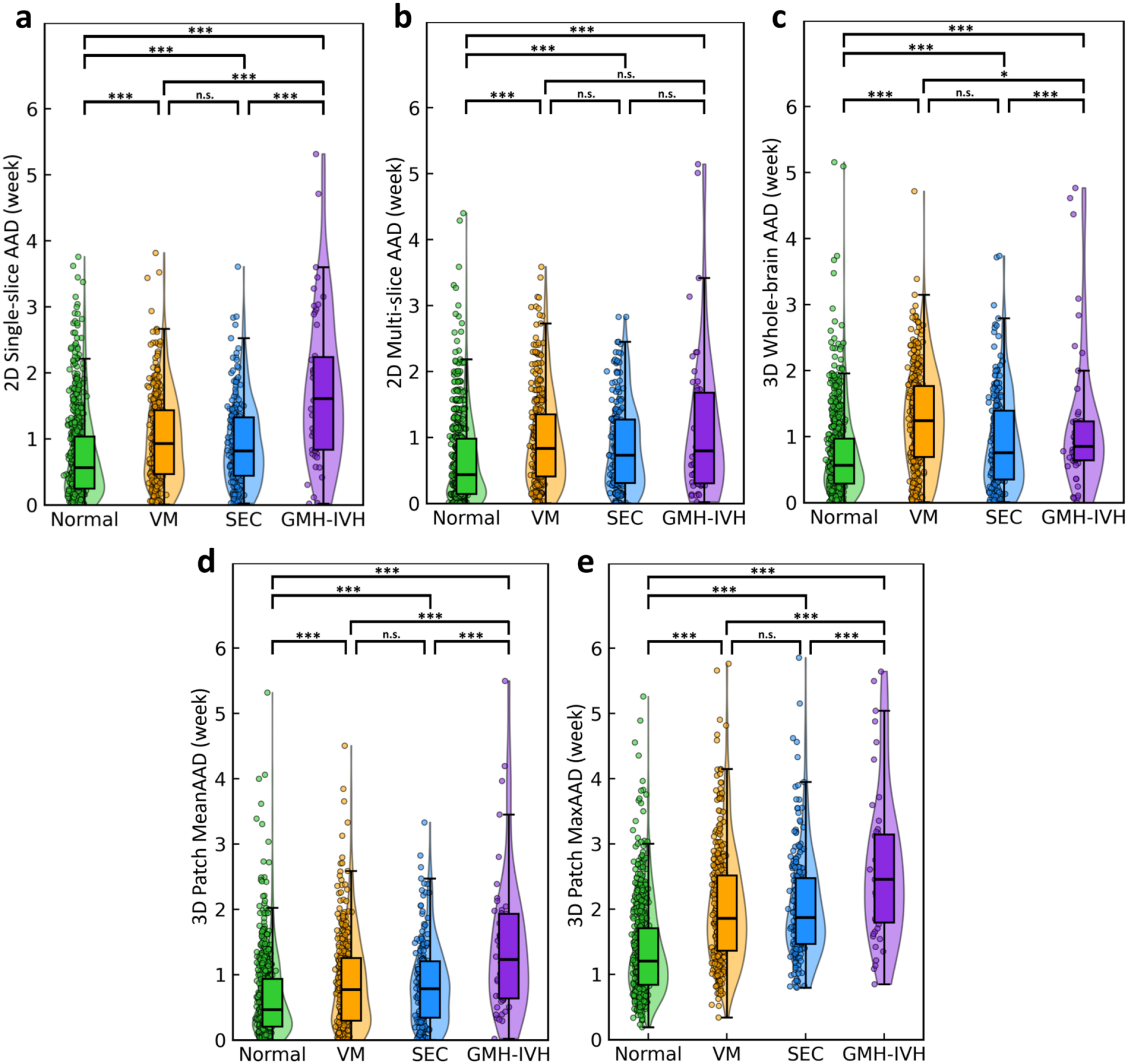
Biomarker value distribution across clinical statuses. The significance levels are represented as follows: n.s. indicates a non-significant result (*P* > 0.05), while *, **, and *** indicate varying levels of statistical significance (0.01 < *P* ≤ 0.05, 0.001 < *P* ≤ 0.01, and *P* < 0.001, respectively).

In addition to improving anomaly detection accuracy, our results also reveal that different anomaly types exhibited distinct MaxAAD patterns (Table 7, Fig. 6). The MaxAAD of GMH-IVH (2.96 ± 1.75 weeks) was significantly greater (*P* < 0.001) than that of VM (2.04 ± 0.93 weeks) and SEC (2.05 ± 0.82 weeks). In contrast, 3D Whole-brain AAD and 2D Multi-slice AAD struggled to establish a clear boundary between GMH-IVH and VM/SEC. For instance, 3D Whole-brain AAD of GMH-IVH (1.17 ± 1.09 weeks) was even lower than that of VM (1.26 ± 0.75 weeks). Similarly, 2D Multi-slice AAD showed limited discriminative power, with a slight gap (<0.2 weeks) between GMH-IVH (1.15 ± 1.14 weeks) and VM (0.96 ± 0.72 weeks).

**Table 7.** Quantitative comparison of biomarker values across methods.

	Biomarker value (mean±std)			
Method	Normal (n = 711)	VM (n = 343)	SEC (n = 212)	GMH-IVH (n = 50)
2D Single-slice AAD	0.75 ± 0.73	1.01 ± 0.68	0.93 ± 0.64	1.75 ± 1.16
2D Multi-slice AAD	0.64 ± 0.67	0.96 ± 0.72	0.86 ± 0.66	1.15 ± 1.14
3D Whole-brain AAD	0.72 ± 0.65	1.26 ± 0.75	0.92 ± 0.73	1.17 ± 1.09
3D Patch MeanAAD	0.65 ± 0.64	0.89 ± 0.73	0.84 ± 0.62	1.69 ± 1.66
PANDA	1.37 ± 0.75	2.04 ± 0.93	2.05 ± 0.82	2.96 ± 1.75

The values represent the AAD (in weeks) between PGA and BGA, expressed as mean ± standard deviation. The proposed PANDA framework utilizes the maximum AAD (MaxAAD) across patches, while comparison methods utilize slice-based, whole-brain, or patch-based mean AAD metrics.

VM = ventriculomegaly; SEC = subependymal cysts; GMH-IVH = germinal matrix-intraventricular hemorrhage.

**Fig. 7.**
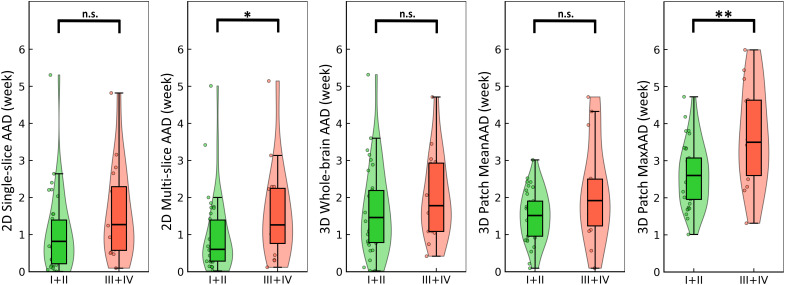
Biomarker value distribution across GMH-IVH grades. The significance levels are represented as follows: n.s. indicates a non-significant result (*P* > 0.05), while *, **, and *** indicate varying levels of statistical significance (0.01 < *P* ≤ 0.05, 0.001 < *P* ≤ 0.01, and *P* < 0.001, respectively).

Moreover, significant differences in MaxAAD were observed between GMH-IVH severity groups using PANDA (*P* < 0.01), with biomarker values increasing from 2.62 ± 0.85 weeks in mild cases (Grade I & II) to 3.66 ± 1.32 weeks in severe cases (Grade III & IV) (Fig. 7, Table 8). In contrast, no significant differences were observed for 2D Single-slice AAD, 3D Whole-brain AAD, or 3D Patch MeanAAD. Quantitative analyses further confirm that PANDA achieved the best performance in differentiating GMH-IVH severities (Table 8). Specifically, PANDA attained the highest diagnostic metrics with an AUROC of 0.725 and an AUPR of 0.636 for distinguishing mild from severe GMH-IVH, outperforming other methods.

**Table 8.**
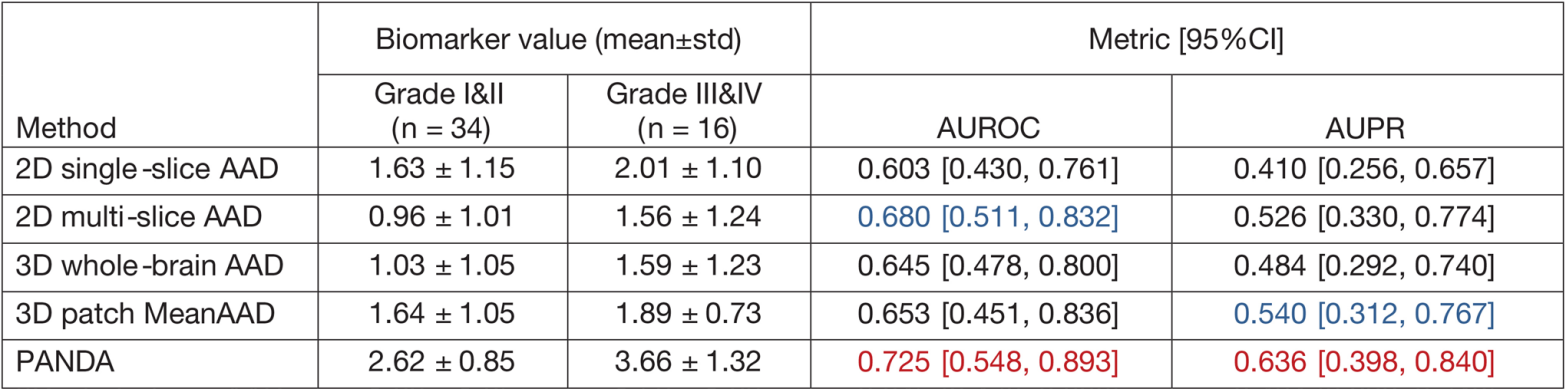
Grading performance of GMH-IVH across models.

The meanings of the colored fonts in the table are as follows: red represents the best performance of the metrics, blue represents the sub-optimal performance of the metric.

GMH-IVH = germinal matrix and intraventricular hemorrhage; AUROC = area under the receiver operating characteristic curve; AUPR = area under the precision recall curve; 95% CI = 95% confidence interval.

### 3.8 Fetal brain age prediction performance

The advantage of 3D patch-based mechanism extends beyond anomaly detection. Specifically, the mean PGA averaged across all patches was more accurate in predicting the GA of normal fetuses (Table 9), achieving an *R²* of 0.922, surpassing 2D Single-slice (0.899), 2D Multi-slice (0.921), and 3D Whole-brain (0.913) methods. Therefore, the mean PGA is recommended for brain age prediction related tasks, while MaxAAD is recommended as a biomarker for brain anomaly detection in practice.

**Table 9.** Brain age prediction performance (the coefficient of determination for linear regression *R^2^*) of different methods.

Method	Normal (n = 711)	VM (n = 343)	SEC (n = 212)	GMH-IVH (n = 50)
2D Single-slice	0.899	0.812	0.830	0.575
2D Multi-slice	0.921	0.829	0.836	0.744
3D Whole-brain	0.913	0.796	0.753	0.744
PANDA (ViT)	0.902	0.754	0.708	0.623
PANDA (V-Mamba)	0.887	0.705	0.688	0.577
PANDA	0.922	0.840	0.849	0.457

VM = ventriculomegaly; SEC = subependymal cysts; GMH-IVH = germinal matrix-intraventricular hemorrhage.

## 4 Discussion

Our proposed fetal brain anomaly detection framework, PANDA, does not require manually annotated anomaly labels, as it is trained exclusively on data from normal fetuses for brain age prediction, substantially reducing annotation costs and enhancing clinical feasibility. However, PANDA’s anomaly detection performance was not compromised and achieved an AUROC of 0.762 and an AUPR of 0.790, significantly outperforming existing 2D slice-based and 3D whole-brain age prediction methods (*P* < 0.05). Subgroup analyses further demonstrated PANDA’s consistent superiority, achieving AUROCs of 0.740, 0.765, and 0.904 for detecting VM, SEC, and GMH-IVH, respectively. Moreover, by utilizing patch-wise MaxAAD as a biomarker, PANDA provided coarse localization of pathological regions.

PANDA’s superior performance stems from its effective incorporation of 3D global information and the simultaneous capability to distinguish patches containing abnormal regions. In comparison, 2D Single-slice AAD analyzes only central slices, losing anomaly information absent from these specific planes from the beginning, while 2D Multi-slice AAD suffers from reduced spatial resolution, potentially obscuring critical 3D structural details. Even though Whole-brain AAD learns PGA from an entire brain volume, it tends to diminish the sensitivity to abnormal brain regions (Hong et al., 2021). Additionally, supervised baselines are prone to overfitting and have limited generalizability, particularly in external validation. This is largely due to their reliance on labeled abnormal cases, which are both scarce and highly heterogeneous in fetal brain MRI.

PANDA’s superior performance is also attributable to its network architecture, patch size selection, and aggregation strategy, as demonstrated by ablation studies (Table 3, Table 9, Fig. 5). The lightweight SFCN outperforms ViT and V-Mamba, whose greater complexity is more prone to overfitting on the limited training set, resulting in lower age prediction accuracy and consequently inferior anomaly detection performance. A patch size of 64 × 64 × 64 voxels provides an optimal balance, as smaller patches lack sufficient developmental landmarks for reliable age estimation, whereas larger patches may dilute focal abnormal signals with surrounding normal tissue. The aggregation strategy interacts with the patch size 64 × 64 × 64, at which MaxAAD is optimal because each patch contains sufficient contextual information for stable prediction, whereas at 32 × 32 × 32, top-*k* averaging compensates for the increased number of patches.

The diagnostic capability of PANDA is particularly superior for detecting compound structural anomalies, which are often associated with poorer neurodevelopmental outcomes (Davutoglu et al., 2026), compared with isolated anomalies. Specifically, PANDA achieves exceptional performance in identifying GMH-IVH (AUROC = 0.904), outperforming its ability to detect VM (AUROC = 0.740) and SEC (AUROC = 0.765). Additionally, the MaxAAD for GMH-IVH was significantly greater than that for VM and SEC. The likely explanation for this disparity is that GMH–IVH involves both ventricular dilation (with VM present in grade II–IV cases, 45 of 50 in our study) and additional parenchymal destruction, accompanied by distinct intensity changes in T_2_-weighted images caused by hemorrhage (Epstein et al., 2021; Sanapo et al., 2017). In addition, our result is consistent with a previous study which also demonstrated AAD can effectively differentiate isolated VM from VM with associated central nervous system (CNS) anomalies (Yun et al., 2025). Furthermore, the clinical utility of PANDA is highlighted by its superior ability to differentiate GMH-IVH severity, which is of critical clinical importance given the significant difference in outcomes. Mild cases (Grade I/II) typically indicate a favorable prognosis, whereas severe cases (Grade III/IV) are associated with a 31–57% mortality rate and serious complications such as cerebral palsy, epilepsy, and developmental delays (Dunbar et al., 2021; Epstein et al., 2021; Kim et al., 2024; Moradi et al., 2024; Sanapo et al., 2017; Sileo et al., 2022; Vassar et al., 2024; Wang et al., 2022).

In the broader context of unsupervised fetal brain anomaly detection (Cao et al., 2023), recent advances have leveraged deep generative models, such as Variational Autoencoders (VAEs) and Diffusion Models, to reconstruct normative counterparts of input images (Mykula et al., 2024). For instance, the CCVAEGAN framework (You et al., 2025), which employs covariate-conditioned encoding and cycle-consistency training, was able to distinguish between normal and anomalous cases. Diffusion model-based approaches, such as iNAAD (Olsen et al., 2024), have also been applied to fetal brain ultrasound anomaly detection, attaining an AUROC of 0.740 for VM detection. While these generative approaches offer pixel-level anomaly localization via reconstruction error maps, they often require substantial computational resources and complex training stability tuning (Bercea et al., 2023; Chen & Peng, 2024; M. Liu, Gan, et al., 2024). PANDA adopts a discriminative, patch-wise age prediction approach that directly exploits the correlation between structural maturity and chronological age, collectively contributing to SOTA performance. By eliminating the need for fully reconstructed images, it achieves computational efficiency by leveraging GA information. Moreover, PANDA provides a robust and interpretable patch-wise biomarker that reliably identifies pathological regions, improving upon other anomaly detection approaches using either slice-wise or whole-brain age prediction but cannot provide any localization information, striking a balance between model complexity and interpretability. In summary, although PANDA does not produce pixel-level anomaly maps as precisely as reconstruction-based generative models (Ambekar et al., 2024; Bercea et al., 2022, 2025), its computational efficiency enables more efficient and clinically accessible disease diagnosis, together with coarse patch-wise interpretability.

PANDA may be applicable to many other abnormalities beyond those investigated in this work (i.e., VM, SEC, and GMH-IVH). For example, PANDA holds promise for diagnosing other anomalies that alter key brain structures essential for gestational age prediction, such as the disruption of the cerebellum and vermis in Dandy-Walker malformation (DWM) (Akiyama et al., 2022) and the alteration of midline commissural structures in agenesis of the corpus callosum (ACC) (Santo et al., 2012). Additionally, PANDA has the potential to detect non-structural abnormalities, particularly in cases of fetal growth restriction (FGR). Unlike reconstruction-based methods that primarily target localized structural defects, PANDA can capture FGR because it induces global reductions in cortical and cerebellar volumes, thereby slowing the overall brain maturation rate. However, the sensitivity of PANDA may decrease if structural abnormalities are located in regions that contribute less to the gestational age prediction model (Shi et al., 2020), such as ventricular contours and parenchymal boundaries.

There are a few limitations to our study. (1) PANDA is unable to provide pixel-level anomaly maps for precise lesion delineation. Future work could explore integrating PANDA with generative models, such as lightweight autoencoders or efficient diffusion models, to achieve finer-grained anomaly localization while preserving computational efficiency and clinical applicability. (2) The robustness of PANDA has been validated on three abnormality types (i.e., VM, SEC, and GMH-IVH), and its generalizability to other fetal brain anomalies remains to be further established. Future studies should evaluate PANDA on a broader spectrum of pathologies, including non-structural conditions such as FGR and structural malformations such as DWM and ACC. (3) The current validation datasets exhibit an imbalance in magnetic field strength, with abnormal cases predominantly acquired with 1.5-T scanners. Future work should incorporate more balanced multi-site, multi-field-strength datasets to further evaluate the robustness of PANDA across diverse imaging protocols.

## 5 Conclusion

In conclusion, this study demonstrates the diagnostic power of the 3D patch-based framework PANDA for detecting a range of fetal brain anomalies. By leveraging patch-wise MaxAAD as a biomarker, PANDA not only significantly outperforms slice- and whole-brain age prediction methods in identifying VM, SEC, and GMH-IVH, but also provides interpretable, localized insights into pathological regions. This robust and fully automated tool holds substantial potential to support clinicians in the early, accurate, and comprehensive detection of fetal neuropathologies, ultimately contributing to improved prenatal care and informed clinical decision-making, while also enabling patch-level assessment of both normal and atypical fetal brain development. By capturing subtle developmental deviations even in regions without overt lesions, PANDA provides a valuable framework for advancing our understanding of normal and abnormal neurodevelopmental processes.

## Data Availability

Data generated or analyzed during the study are not publicly available, but are available from the corresponding author by request. The source codes of PANDA are implemented using Pytorch, which are available at: https://github.com/birthlab/PANDA
